# Engineering the surface properties of a human monoclonal antibody prevents self-association and rapid clearance *in vivo*

**DOI:** 10.1038/srep38644

**Published:** 2016-12-20

**Authors:** Claire L. Dobson, Paul W. A. Devine, Jonathan J. Phillips, Daniel R. Higazi, Christopher Lloyd, Bojana Popovic, Joanne Arnold, Andrew Buchanan, Arthur Lewis, Joanne Goodman, Christopher F. van der Walle, Peter Thornton, Lisa Vinall, David Lowne, Anna Aagaard, Lise-Lotte Olsson, Anna Ridderstad Wollberg, Fraser Welsh, Theodoros K. Karamanos, Clare L. Pashley, Matthew G. Iadanza, Neil A. Ranson, Alison E. Ashcroft, Alistair D. Kippen, Tristan J. Vaughan, Sheena E. Radford, David C. Lowe

**Affiliations:** 1 MedImmune Ltd, Granta Park, Cambridge, CB21 6GH, UK; 2 Astbury Centre for Structural Molecular Biology, University of Leeds, Leeds, LS2 9JT, UK; 3 School of Molecular and Cellular Biology, University of Leeds, Leeds, LS2 9JT, UK; 4 Department of Chemical Engineering and Biotechnology, University of Cambridge, Cambridge, CB2 3RA, UK; 5 Discovery Sciences, Innovative Medicines and Early Development, AstraZeneca, Pepparedsleden 1, Mölndal, 43183, Sweden.

## Abstract

Uncontrolled self-association is a major challenge in the exploitation of proteins as therapeutics. Here we describe the development of a structural proteomics approach to identify the amino acids responsible for aberrant self-association of monoclonal antibodies and the design of a variant with reduced aggregation and increased serum persistence *in vivo.* We show that the human monoclonal antibody, MEDI1912, selected against nerve growth factor binds with picomolar affinity, but undergoes reversible self-association and has a poor pharmacokinetic profile in both rat and cynomolgus monkeys. Using hydrogen/deuterium exchange and cross-linking-mass spectrometry we map the residues responsible for self-association of MEDI1912 and show that disruption of the self-interaction interface by three mutations enhances its biophysical properties and serum persistence, whilst maintaining high affinity and potency. Immunohistochemistry suggests that this is achieved via reduction of non-specific tissue binding. The strategy developed represents a powerful and generic approach to improve the properties of therapeutic proteins.

Monoclonal antibodies (mAbs) represent the fastest growing class of therapeutics in the pharmaceutical sector, with over 50 therapeutic antibodies currently on the market^1^. The success of antibodies as drugs can be attributed to their high binding affinity and exquisite specificity, combined with low intrinsic toxicity. Therapeutic antibodies are usually administered via intravenous infusion or subcutaneous injection. A long circulating half-life *in vivo,* therefore, is highly desirable to reduce the frequency of antibody administration, improving patient compliance and clinical benefit. Antibodies with unexpectedly rapid plasma clearance due to off-target mediated elimination mechanisms have been observed in preclinical studies with rats and cynomolgus monkeys^2^,^3^. Rapid clearance in preclinical models has been shown to correlate with similar observations in the clinic^4^ thus compromising the therapeutic utility of the antibody. Efforts have been made to correlate plasma clearance rates with sequence-determined characteristics of the antibody and to develop *in silico* or *in vitro* screens to predict this behaviour^5^,^6^,^7^.

Therapeutic antibodies must be able to withstand a range of stresses during manufacture. These include variations of temperature, pH, ionic strength, exposure to air-water interfaces, high protein concentrations and mechanical strain, any of which may affect the stability of the antibody^8^. Protein aggregation involving reversible self-association is an increasingly recognised problem affecting the bioprocessing of human therapeutic antibodies that influences both shelf life and efficacy^9^,^10^,^11^. This has become more common due to a growing trend towards formulations that allow sub-cutaneous administration routes. As a consequence, large amounts of antibody (>100 mg) must be delivered in a single administration of a relatively small volume (<2 mL), resulting in the need for concentrated antibody formulations (>50 mg/mL) in devices for self-administration. This places increased demands on antibody solubility and colloidal stability (the propensity of the folded protein to precipitate)^12^. Monoclonal antibodies that are prone to aggregation may form aggregate structures that appear foreign to a patient’s immune system and therefore elicit an immune response that ablates the therapeutic activity of the administered drug^13^,^14^. Clinical success, therefore, not only requires an appropriate pharmacology and pharmacokinetic profile, but also that the antibody must exhibit appropriate biophysical properties. A variety of computational approaches^6^,^15^,^16^ and simple experimental assays have been developed to identify antibody variants with increased aggregation propensity and decreased colloidal stability^17^,^18^,^19^. Using these tools it is also possible to engineer antibodies with poor solubility or high aggregation propensity to ameliorate these characteristics^20^,^21^,^22^, although this approach can be difficult if the problematic region of the antibody resides in the paratope^15^.

Here we describe MEDI1912, an anti-nerve growth factor (NGF) antibody for the potential treatment of chronic pain that inhibits signalling via the TrkA and p75 receptors. MEDI1912 has a picomolar *K*_*d*_ for NGF, but displays aberrant biophysical and solution properties, as well as an impaired non-linear pharmacokinetic (PK) profile in rats and cynomolgus monkeys, endangering product development. Here, we applied hydrogen/deuterium exchange - mass spectrometry (HDX-MS) and cross-linking MS (XL-MS)^22^,^23^,^24^ combined with negative stain EM to map the self-association interface within MEDI1912. Moreover, we present an approach that combines this with *in silico* aggregation prediction tools to map the specific amino acids responsible for driving antibody self-association, from which we were able to design a triple mutant that disrupts the interaction interface without compromising potency or affinity for antigen (NGF). By improving the biophysical and solution properties of MEDI1912, a concurrent improvement in serum half-life and binding specificity *in vivo* was also achieved, indicating the mutual benefit of improved biophysical behaviour and biological response. The HDX/XL-MS and targeted mutagenesis methodology employed represents a powerful approach that could be used as a generic strategy to improve the robust and reproducible manufacture of antibody-based medicines and protein therapeutics.

## Results

### Characterisation of human monoclonal antibody, MEDI1912

**Figure 1:**
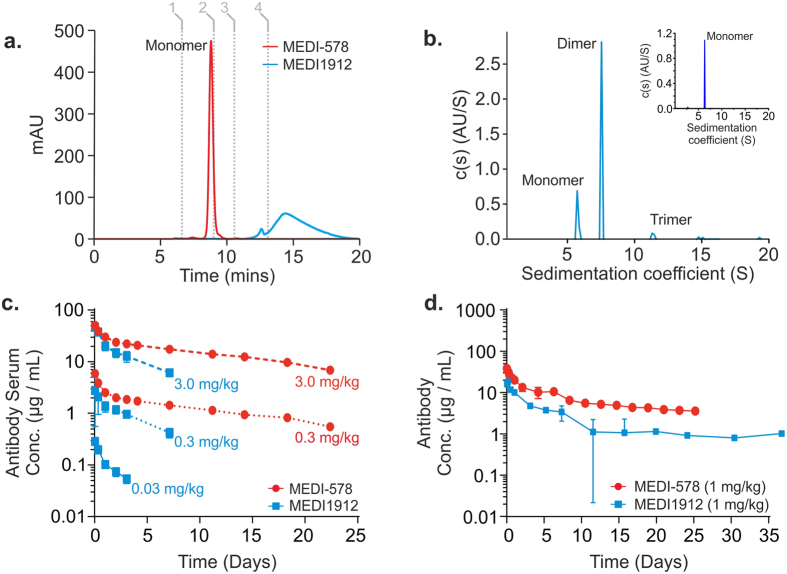
Biophysical and PK properties of MEDI-578 and MEDI1912. (**a**) Size exclusion chromatography-HPLC (SEC-HPLC) elution profiles of MEDI-578 (red) and MEDI1912 (blue), each 1 mg/mL, at 280 nm (mAu) (0.1 M sodium phosphate, 0.1 M sodium sulphate, pH 6.8). Grey lines show calibrants used: Thyroglobulin (1), IgG (2), Ovalbumin (3) and Vitamin B_12_ (4). (**b**) Sedimentation-velocity analytical ultracentrifugation (SV-AUC) of MEDI1912 (1 mg/mL, 20 mM sodium succinate, 125 mM arginine, pH 6). (**c**) MEDI-578 and MEDI1912 PK profiles following intravenous administration of a 3.0 mg/kg, 0.3 mg/kg or 0.03 mg/kg dose to rats (n = 3). (**d**) MEDI-578 and MEDI1912 PK profiles following intravenous administration of a 1 mg/kg dose to cynomolgus monkeys (n = 3). Data points represent the mean ± SD.

MEDI-578 is a phage display derived anti-NGF antibody with a *K*_*d*_ of 69 pM. MEDI1912 was generated by *in vitro* affinity maturation of MEDI-578 and expressed transiently in a Chinese hamster ovary (CHO) cell line. Although IgG expression levels (200 mg/L) were in the range typical for transiently expressed recombinant human IgGs^25^, unlike MEDI578, MEDI1912 showed colloidal instability (protein precipitation, opalescence and phase separation), adsorption to filter membranes, and gelation, resulting in poor yields (<30%) during purification. Consistent with this behaviour during bioprocessing, MEDI1912 exhibited a long retention time and broad asymmetric peak shape when analysed by high performance size exclusion chromatography (HP-SEC) (Fig. 1a), indicating non-specific binding to the SEC column matrix^19^. Sedimentation-velocity analytical ultracentrifugation (SV-AUC) revealed that MEDI1912 (1 mg/mL in formulation buffer (Methods)) self-associates to form dimers with small amounts of higher order oligomers (Fig. 1b), while dynamic light scattering (DLS) showed that MEDI1912 formed oligomers under all buffer conditions tested in a concentration-dependent manner (Supplementary Figure 1a). MEDI1912 also exhibited low solubility (Supplementary Figure 1b) and antibody solutions were highly viscous (Table 1) even at low concentrations (9.8 mg/mL). None of these undesirable properties was observed for MEDI-578 (data not shown).

We next performed pharmacokinetic studies on MEDI1912 and MEDI-578. Following intravenous administration to healthy rats and cynomolgus monkeys, MEDI1912 demonstrated a ~3-fold shorter half-life and a faster rate of clearance compared with MEDI-578 in both species (17.90 ± 5.99 (SD) mL/kg per day for MEDI1912 vs. 4.74 ± 0.77 (SD) mL/kg per day for MEDI-578 in cynomolgus monkeys at 1 mg/kg dose; 25.11 mL/kg per day for MEDI1912 vs. 7.07 mL/kg per day for MEDI-578 in rats at 3 mg/kg dose) (Fig. 1c,d, and Supplementary Table 1a,b).

### Mapping MEDI1912 self-interactions

**Figure 2:**
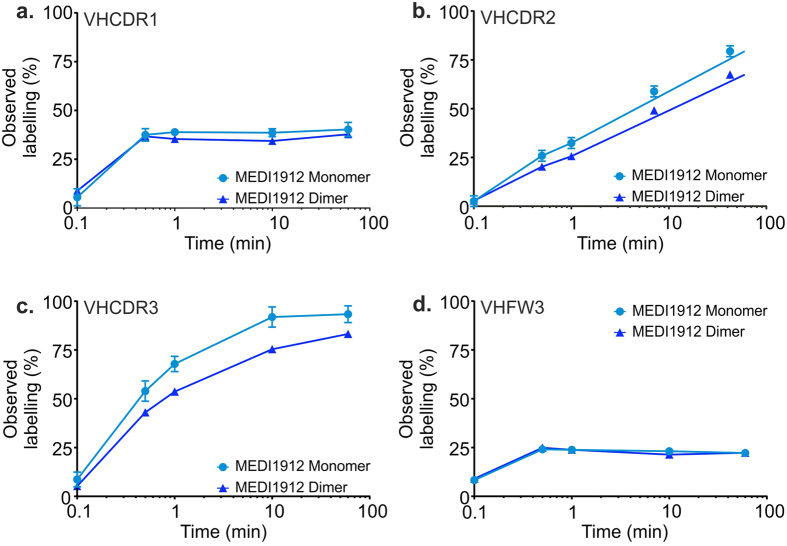
MEDI1912 dimer shows site-specific protection against hydrogen exchange. HDX-MS identified peptide regions within MEDI1912 that have a lower rate of labelling under conditions that stabilise the antibody dimer (1 mg/mL) versus the monomer (0.1 mg/mL). Significant differences in labelling (p < 0.01) were observed in VHCDR1 (**a**), VHCDR2 (**b**) and VHCDR3 (**c**), yet there was no difference, for example, in a peptide within VH framework region 3). This supports the hypothesis that there is a specific interface for MEDI1912 self-association involving the variable domains. Data are corrected for back-exchange and represent mean ±1 SD.

**Figure 3:**
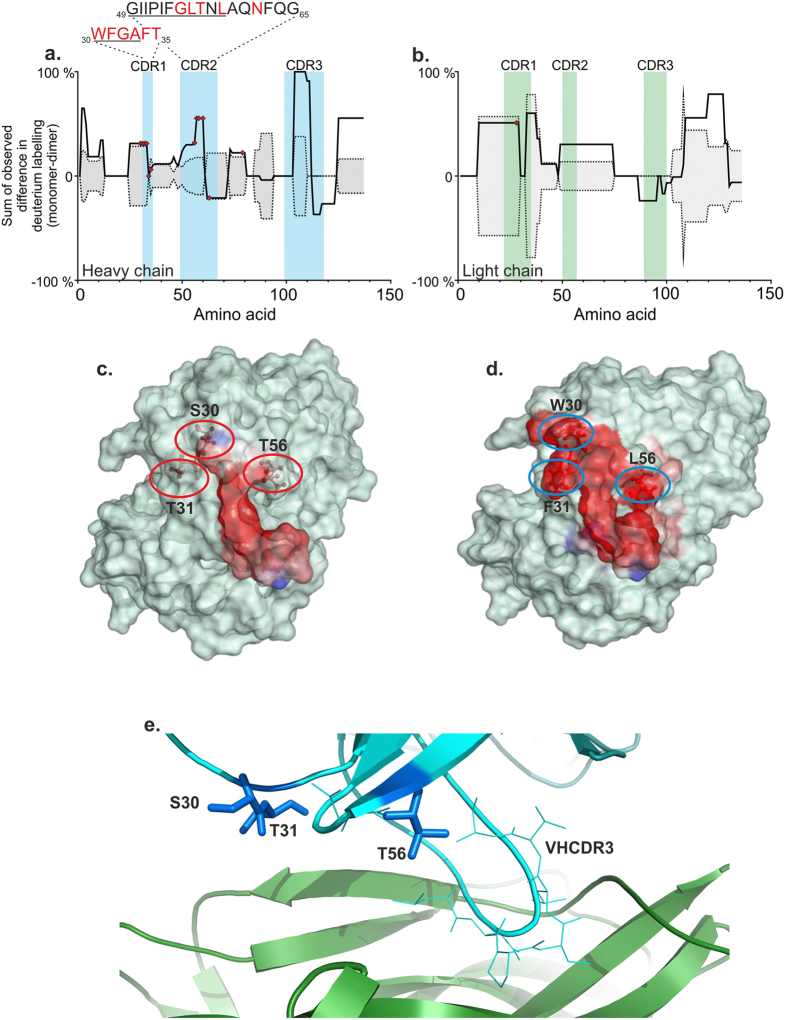
Mapping MEDI1912 self-interactions. Site-localised protection against hydrogen-exchange in MEDI1912 heavy (**a**) and light (**b**) chain variable domains. For concentrations at which MEDI1912 is predominantly monomeric (M) and dimeric (D), the difference in uptake of deuterium label was calculated as ΔHDX_(M-D)._ This was normalised per protein, with the region that showed maximum difference set to 100%. Protection due to self-association shows as a positive value on the y-axis (solid line – measured value; dashed line – 99% confidence limit). Amino acids altered in affinity maturation from MEDI-578 are marked in red circles. CDR sequences are listed for those that were affinity matured: red – mutated residue; underlined – significant HDX protection (p < 0.01). A spatial aggregation propensity (SAP) algorithm was applied to structural models of MEDI-578 (**c**) and MEDI1912 (**d**) revealing surface exposed hydrophobic patches (red)^15^. Three amino acids coincident with significant HDX protection in MEDI1912 dimer and SAP prediction: W30, F31 and L56 (blue circles) were chosen for reversion to the corresponding MEDI-578 amino acids: S30, T31, and T56 (red circles). The crystal structure of NGF (coloured green) in complex with the Fab of the MEDI1912 parental antibody, MEDI-578 (blue), (**e**) reveals that while the VHCDR3 (cyan lines) forms a loop that extends into a cavity within NGF, the amino acid residues 30 and 31 in the VHCDR1 and 56 in the VHCDR2 (blue sticks) are located at the periphery of the binding interface and do not make specific interactions with epitope residues.

To identify the region of MEDI1912 responsible for these poor biophysical and pharmacokinetic properties, hydrogen/deuterium-exchange mass spectrometry (HDX-MS) was performed under solution conditions^24^,^26^,^27^ in which the antibody is either predominantly dimeric (>75% dimer by SV-AUC at 1.0 mg/mL) or monomeric (0.1 mg/mL). All three VH complementarity determining regions (CDRs) were identified as exhibiting significant differences (pairwise Student t-test p < 0.01) in their uptake of deuterium label upon dimerisation (Fig. 2). The deuterium labelling for these peptides can be mapped to the antibody primary sequence, permitting sub-molecular localisation of the changes. A subtraction of the two measurements, ΔHDX_(M-D)_, identifies regions in which there was a change in structural dynamics upon dimerization (Fig. 3a,b). In studies of self-association, the dissociation rate can impact kinetic studies; therefore in the present study we were careful to analyse relative differences in deuterium labelling and with a time course orders of magnitude greater than the predicted half-life of the dimer. Several sites were implicated in MEDI1912 dimerisation with significantly increased protection upon dimer formation. The highest degree of relative protection (globally normalised to ΔHDX_(M-D)_ = 100%) was observed in the CDR3 loop of the V_H_ domain (VHCDR3); residues Asp100-Leu100e defined by Kabat *et al*.^28^. However, no changes were made in this region during affinity maturation, suggesting that this region may have an intrinsic propensity to drive self-association, but requires additional neighbouring areas to form a stable dimer interface. Additional significant protection was observed in three regions in the dimer which contain mutations from affinity maturation: VHCDR2 and VHCDR3, while VHCDR1 showed a marginal (but statistically significant) increase in protection. Nine overlapping peptides were identified that map to VHCDR2 (residues Gly50-Gly65), yielding high resolution information about which amino acids show hydrogen/deuterium exchange protection in the dimers/oligomers. This enabled the identification of residues which have significantly increased protection in the IgG dimer to Gly50-Leu59 (Supplementary Figure 2). Four of these amino acids (Gly55, Leu56, Thr57 and Leu59) were mutated (from Asp55, Thr56, Gly57 and Ser58) in the affinity maturation of MEDI-578 to MEDI1912. The same approach, when applied to VHCDR1 (residues Phe31-Thr35), revealed significant protection in Ala24-Ala33, but not in Phe34-Thr35. Together, these data indicate which of the mutated amino acids co-localise with sites of significantly increased hydrogen/deuterium exchange protection. Nine amino acids in all were found to be potentially responsible for dimer formation in MEDI1912: Trp30, Phe31, Gly32, Ala33, Gly55, Leu56, Thr57, Leu59 and Val78 – all in the heavy chain.

To visualise the aggregation-prone regions of MEDI1912, a structural model of the IgG was built using the crystal structure of NGF in complex with MEDI-578 Fab as a template (pdb 5jz7). A spatial aggregation propensity (SAP) algorithm^15^ (Accelrys) was then applied to the MEDI-578 (Fig. 3c) and MEDI1912 (Fig. 3d) antibody models to identify surface exposed hydrophobic patches. The aggregation-prone site for MEDI1912 predicted by SAP was found to be consistent with that determined by HDX-MS. By analysing both data sets, three exposed hydrophobic residues unique to MEDI1912 were identified at positions W30, F31 and L56. The structure also reveals that while the VHCDR3 forms a loop that extends into a cavity within NGF, W30, F31 and L56 are located at the periphery of the binding interface and do not make specific interactions with residues in the epitope (Fig. 3e).

### Engineering the MEDI1912 interface to disrupt self-interaction

**Figure 4:**
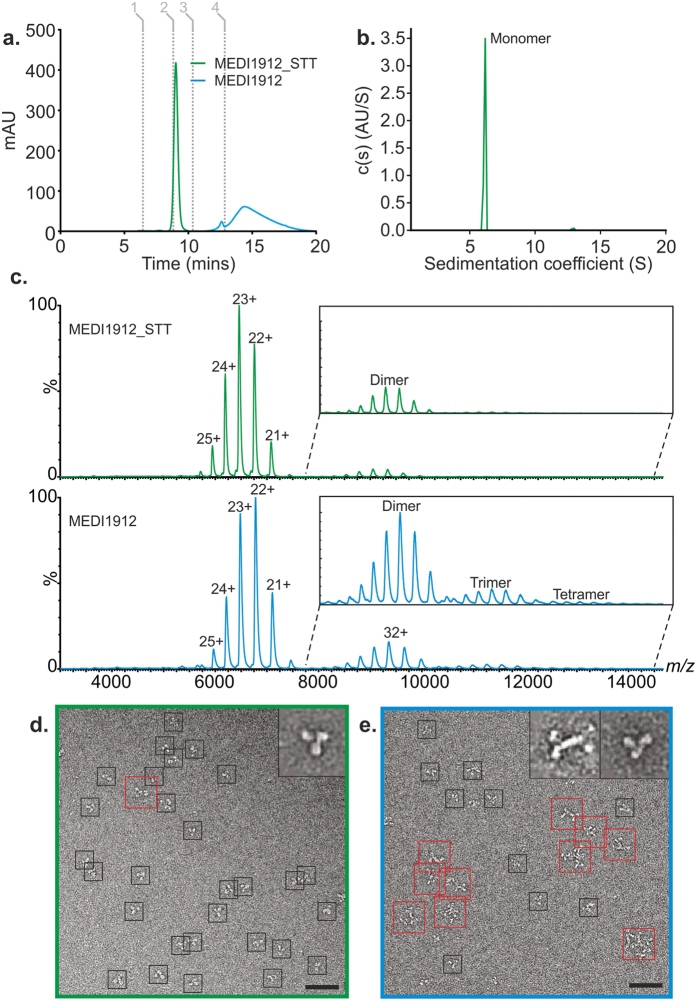
Conformational properties of MEDI1912 and MEDI1912_STT using nESI-IMS-MS and negative stain TEM. (**a**) High performance size exclusion chromatography-HPLC (HP-SEC-HPLC) elution profiles of MEDI1912 (blue) and MEDI1912_STT (green) at 280 nm (mAU). Grey lines show calibrants used: Thyroglobulin (1), IgG (2), Ovalbumin (3) and Vitamin B_12_ (4). (**b**) SV-AUC of MEDI1912_STT showing the distribution of species formed. (**c**) Nano electrospray ionisation (nESI) mass spectra of MEDI1912 (blue, lower) and MEDI1912_STT (green, upper) showing the different oligomers observed for the two proteins. The insets highlight the higher-order species formed from each sample. (**d**) Negative stain TEM images of MEDI1912_STT (green, left) and MEDI1912 (blue, right) reveal a distribution of oligomeric species only for the latter protein. Black boxes, monomer; red boxes, dimers/oligomers. Scale bar = 25 nm. Inset shows averaged particle images for monomer and dimer species observed (31 nm (square)).

To reduce the self-association of MEDI1912, we reverted the three key hydrophobic residues (W30, F31 and L56), either individually or in combination, to the corresponding amino acids in the parental MEDI-578. The variants were subsequently tested as purified IgGs by HP-SEC to assess whether non-specific binding of MEDI1912 to the column (an indicator of self-assembly, poor colloidal stability and solubility) had been ameliorated. Each of the variants exhibited improved chromatographic resolution and a shorter retention time compared with MEDI1912 (Table 2). The most improved variant, MEDI1912_STT, combining the mutations W30S, F31T and L56T, resulted in a single peak with a monomer retention time (8.9 minutes) similar to that of the reference and parental IgGs (Fig. 4a; Table 2). Consistent with these measurements, DLS experiments showed that MEDI1912_STT did not oligomerise (at concentrations from 1–6 mg/mL) under all buffers conditions tested (Supplementary 1c). MEDI1912_STT also exhibited improved relative solubility (Supplementary Figure 1b) and solution viscosity (Table 1). Furthermore, protection against hydrogen-exchange, due to self-association, is diminished in MEDI1912_STT relative to MEDI1912 (Supplementary Figure 3).

Native nanoelectrospray ionisation-mass spectrometry (nESI-IMS-MS) was next performed to probe the self-association of MEDI1912 and MEDI1912_STT. nESI-MS allows the solution structures of covalently and non-covalently bound protein complexes to be preserved in the transition to the gas phase and information about their conformational properties can be revealed from both mass and charge state distribution^29^,^30^. HP-SEC, AUC and DLS revealed that MEDI1912_STT is predominantly (>98%) monomeric (at 1 mg/mL) in both formulation and mass spectrometry-compatible buffers, while MEDI1912 oligomerises under both conditions, forming dimers and higher order species (Figs 1b and 4a,b and Supplementary Figure 1c). nESI-MS confirmed the improved properties of MEDI1912_STT, revealing predominantly monomers (expected mass = 148,107.76 Da, measured mass = 148,119.45 ± 8.98 Da) together with 2.8 ± 0.3% dimer (estimated with respect to the monomer ion intensity) (Fig. 4c). By contrast, monomers (expected mass = 148,421.96 Da, measured mass = 148,430.13 ± 6.82 Da), combined with oligomers up to, and including, tetramers are observed in the mass spectrum of MEDI1912, with the dimeric species accounting for 11.4 ± 0.6% of the monomer ion intensity (Fig. 4c). Whilst AUC data shows that MEDI1912 is >~75% dimer at 1 mg/mL, a <20% of dimer is observed in the mass spectrum. This is consistent with the hydrophobic nature of the interactions driving the self-association which are known to be weakened upon transition into the gas-phase^31^,^32^. The MEDI1912 dimer is therefore likely to undergo a degree of dissociation during the ionization process.

To investigate the structural morphology of the oligomeric species formed by MEDI1912 in more detail, the samples were analysed using negative stain transmission electron microscopy (TEM) (Fig. 4d). The results indicated that MEDI1912_STT exists predominantly as monodisperse antibodies, with the expected “Y” shape (Fig. 4d). By contrast, MEDI1912 shows an ensemble of oligomeric structures including dimers (Fig. 4d) consistent with assembly via Fab-Fab interactions. Higher order structures of MEDI1912 are also observed involving entangled polymer species of varying sizes.

To identify the oligomer interface directly, chemical cross-linking (XL) followed by mass spectrometry (XL-MS) was employed. MEDI1912 and MEDI1912_STT were cross-linked at a concentration of 1 mg/mL using a combination of deuterated and non-deuterated BS3 cross-linker. The resulting cross-linked mixtures were separated by SDS-PAGE (Supplementary Figure 4a) and the corresponding dimer bands were excised for in-gel digestion along with cross-linked and non-cross-linked monomer bands. Using the cross-linked monomer band for a comparative analysis, a unique peptide was identified from the 1912 dimer sample (Supplementary Figures 4b–e, 5 and 6a). This unique cross-link was identified as connecting the N-terminus of the heavy chain to K54 in the variable region of the light chain. To generate a model of the dimer interface, data acquired from the SAP algorithm, HDX, knowledge of where the three mutations from MEDI1912 to MEDI1912_STT are positioned and the unique cross-link identified were used to guide docking using HADDOCK (Supplementary Figure 6a) (see Methods). The model generated indicates assembly through Fab-Fab interactions (supplementary Figure 6b), with residues 30, 31 and 56 creating the binding interface, dominated by hydrophobic interactions entirely consistent with the EM data (Fig. 4e). Interestingly, only a single cross-linked peptide was identified using BS3. Analysis of the model structure generated showed that whilst there are 88 lysines in MEDI1912, only two amines (the N-terminus of the heavy chain and K54 of the light chain) are in close enough proximity to be cross-linked (≤11.4 Å), reinforcing the validity of the structural model proposed.

### Pharmacokinetic Profiling of MEDI1912_STT

**Figure 5:**
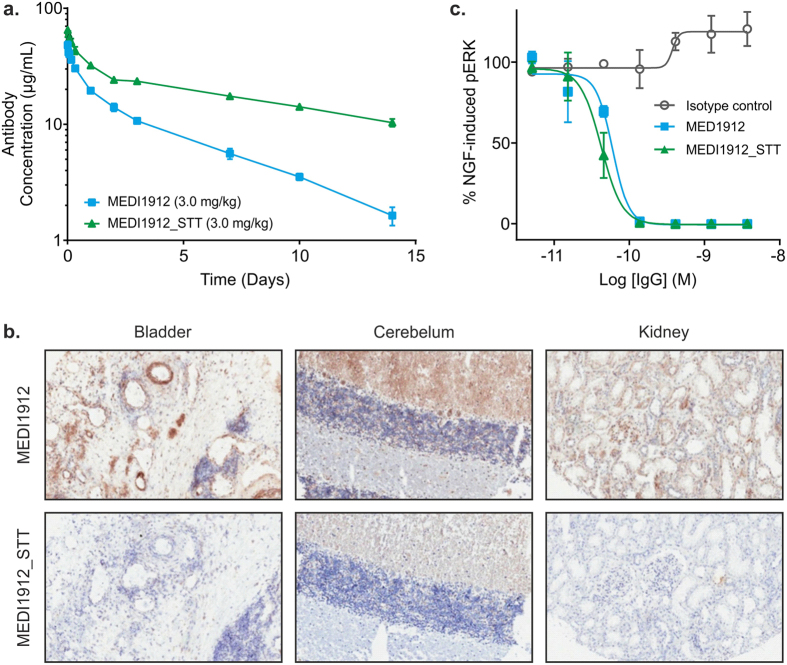
Effect of STT mutations on MEDI1912 pharmacokinetics, tissue specificity and functional potency. (**a**) MEDI1912 and MEDI1912_STT PK profiles following intravenous administration of a 3 mg/kg dose to rats (n = 3). (**b**) Human bladder, cerebellum and kidney tissue immunohistochemically stained with MEDI1912 and MEDI1912_STT at 0.18 μg/mL. Significant staining was demonstrated by MEDI1912, showing strong staining in connective tissue, smooth muscle (around blood vessels) and other areas that is consistent with non-specific staining. MEDI1912_STT showed no evidence of staining in any tissues evaluated. (**c**) MEDI1912_STT retains functional potency *in vitro* compared with MEDI1912 in a phospho-ERK activation assay. Data points represent the mean ± SD.

To investigate the hypothesis that the self-association and the increased serum clearance associated with MEDI1912 are driven by the same molecular properties, MEDI1912 and MEDI1912_STT were administered intravenously to rats at a dose of 3 mg/kg and the antibody serum concentrations were then subsequently measured at various time points. MEDI1912_STT demonstrated a 2-fold improved half-life compared with MEDI1912 (9.43 ± 0.40 (SD) days vs. 4.03 ± 0.40 (SD) days; n = 3), with a reduced clearance rate of 7.26 ± 0.26 (SD) mL/kg/day compared to 24.5 ± 1.40 (SD) mL/kg/day (Fig. 5a and Supplementary Table 1c). To understand whether the rapid clearance of MEDI1912 is mediated through its non-specific association to tissues *in vivo*, we tested binding of MEDI1912 and MEDI1912_STT to a panel of normal human tissue samples using immunohistochemistry (IHC). MEDI1912 displayed significant association to all tissues tested, but no such association was observed for MEDI1912_STT (Fig. 5b and Supplementary Figure 7). The introduction of the STT mutations into the VHCDR1 and VHCDR2 regions of MEDI1912 resulted in no loss in affinity for its antigen (*K*_*d*_ MEDI_1912 = 1.6–9.8 pM; *K*_d_ MEDI1912 STT = 1.8–8.3 pM) (Supplementary Table 2) or decrease in functional potency *in vitro* (Fig. 5c). Together, the results demonstrate that the aberrant biophysical and PK characteristics of MEDI1912 are directly linked and that both can be entirely ameliorated by the retro-engineering of the same three solvent exposed residues without compromising its pharmacological activity or potency.

### Specificity Analysis of MEDI1912

**Figure 6:**
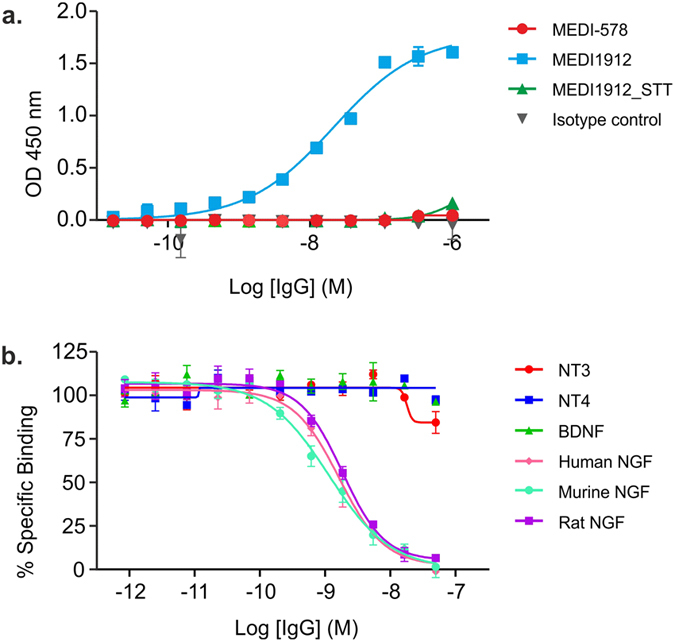
*In vitro* characterisation of MEDI1912 and MEDI1912_STT. (**a**) Baculovirus ELISA and (**b**) specificity of MEDI1912 for human, mouse and rat NGF, and a panel of structurally related proteins determined using a homogeneous time resolved fluorescence assay (HTRF).

In order to further investigate the non-specific tissue association observed with MEDI1912 but not its STT variant, both antibodies, along with parental MEDI578, were assessed in a baculovirus binding assay that has been reported to provide correlation with increased clearance *in vivo*^5^. MEDI1912, but not MEDI1912_STT or MEDI578 exhibited strong binding in this assay (Fig. 6a), further indicating that the three amino acids identified for reversion in MEDI1912 are responsible for both its increased propensity for self-association and the non-specific tissue association that we propose leads to rapid serum clearance *in vivo*. In order to determine whether MEDI1912 had lost binding specificity for human NGF, a homogeneous competition binding assay was performed against human, mouse and rat NGF, along with the closely related proteins neurotrophin 3 (NT3), neurotrophin 4 (NT4) and brain derived neurotrophic factor (BDNF) (Fig. 6b). MEDI1912 showed binding only to human NGF, matching parental MEDI578 (data not shown), indicating that it had maintained its binding specificity to its cognate antigen, despite its increased non-specific association in either the baculovirus particle assay or by immunohistochemistry.

## Discussion

While the generation of high affinity protein therapeutics offers exciting opportunities for the treatment of human disease, realising these opportunities is challenged when the protein therapeutic of interest has an increased propensity to self-associate rendering it aggregation-prone especially under the rigours of bioprocessing^33^, and under storage at the high concentrations required for therapeutic use^34^. Additionally, there is a growing realisation that different antibodies of the same isotype can demonstrate very different pharmacokinetics in both preclinical and clinical studies^5^. Here, we demonstrate that subtle differences in CDR sequences of closely related antibodies can lead to profound differences in their biophysical properties and PK profiles. The anti-human NGF monoclonal antibody MEDI578 did not show significant self-association during its development and as such, represented what we considered to be a molecule suitable for large-scale production. However, in order to achieve a potentially favourable dosing regime in patients, we proceeded to increase its affinity further via *in vitro* affinity maturation, leading to the generation of the higher affinity variant MEDI1912. Whilst binding tightly (*K*_*d*_ 1–10 pM) and specifically to its target antigen, MEDI1912 displayed self-association and poor drug characteristics: colloidal instability, poor solubility, high solution viscosity and an increased aggregation propensity, as well as decreased half-life in serum when administered to rats or cynomolgus monkeys. Transmission electron microscopy confirmed that MEDI1912 does indeed self-associate to form oligomeric species at low protein concentrations. In an attempt to rectify these aberrant properties we used HDX-MS to determine the likely sites of self-interaction. By combining these data with predictions of residues involved in aggregation using the SAP algorithm^15^, three residues potentially contributing to the self-association were identified in the antibody CDRs. Generation of a variant with these three amino acid substituted dramatically improved the antibody, with MEDI1912_STT exhibiting reduced self-association, improved biophysical stability and PK properties with no discernible loss in affinity or biological potency.

Previous examples of antibody re-engineering to improve stability have reported significant reductions in affinity when the problematic residues are located in the CDRs^15^,^20^,^35^. Analysis of the crystal structure of MEDI1912’s parental antibody, MEDI-578, in complex with NGF indicates, however, that the three residues described here do not interact directly with NGF and hence their substitution does not impact binding affinity. These three mutations were most likely neutral mutations that were carried over during the affinity maturation of MEDI578 alongside other amino acid substitutions that do improve binding to antigen.

MEDI1912 displayed an increased propensity to self-associate, as well as rapid clearance in both rodents and non-human primates, rending it unsuitable for further development as a therapeutic. Many recent studies have investigated the causes of rapid antibody clearance^5^,^7^,^36^,^37^, with factors such as pI of the antibody^36^,^38^, increased binding to the neonatal Fc receptor (FcRn) *via* the charge distribution of the Fv domain^37^, non-specific^3^,^39^ or specific off-target binding *in vivo*^40^ all being implicated in poor antibody half-life *in vivo*. Simple *in vitro* assays have been developed to identify antibodies that display non-specific off-target binding, either utilising binding to baculovirus particles (BVP)^5^ or to a yeast-derived soluble membrane protein polyspecificity reagent (PSR)^41^. MEDI1912 and its STT variant were assessed in the BVP binding assay, with MEDI1912, but not MEDI-578 or MEDI1912_STT exhibiting strong binding (Fig. 6a), providing further validation that this assay is indeed capable of identifying antibodies with rapid *in vivo* clearance.

Most strikingly, the results presented show that the same three amino acids responsible for increased self-association of MEDI1912 are also responsible for non-specific association to tissues, when assessed by IHC. This association is likely to be hydrophobically driven, given that the three reversion mutations to produce MEDI1912_STT (and to remove this non-specific binding profile) are Trp → Ser, Phe → Thr and Leu → Thr. Interestingly, MEDI1912 did not appear to bind to the closely related purified proteins in a homogeneous competitive binding assay, indicating that it maintained its binding specificity for human NGF. This demonstrates the importance of screening for non-specific binding in a more complex system, using immunohistochemistry or by binding to complex antigens such as BVPs or PSR. In a recent report, Sharma *et al*. also postulate that the hydrophobicity of antibody light chain CDR1 (L1) and CDR3 (L3) and heavy chain CDR3 (H3) sequences is correlated with high *in vivo* clearance^6^. They describe a simple method whereby the sum of the hydrophobicity index (HI) for these sequences can be correlated with high clearance if this value greater than 4. Using this methodology we calculated that MEDI1912 has a hydrophobicity index (HI) for the sum of LC1, LC3 and HC3 of 5.17, which was determined to be above the threshold (>4) for correlation with a high clearance rate. However, as the three STT mutations lie outside of LC1, LC3 and HC3, MEDI1912_STT would also be predicted to exhibit rapid clearance using this methodology, indicating that such sequence-based methods for predicting PK should be used with caution.

The work described here represents a clear link between self-association of an antibody with poor PK and tissue specificity, and shows that this can be reversed by the introduction of just three amino acid substitutions at solvent-accessible sites identified using HDX-MS and *in silico* aggregation prediction. The residues implicated are close to, but not directly involved, in antigen binding and hence the amino acid substitutions introduced have no effect on affinity, nor do they alter specificity of binding. The combination of HDX/XL-MS and SAP thus represents a powerful approach that could be applied generically to direct engineering of other protein biologics to improve their biophysical and solution properties. The data presented here also suggests that improvement of these *in vitro* characteristics can correlate with improved half-life and PK *in vivo.* Analysis of the properties of antibodies using HDX/XL-MS early in the development pipeline thus promises dramatic improvement in the success rate of developing those that are manufacturable and exhibit pharmacokinetics that are suitable for clinical development.

## Methods

### Affinity maturation of MEDI-578

MEDI1912 was generated by *in vitro* affinity maturation of MEDI-578 via targeted and random mutagenesis of the CDRs using phage and ribosome display, essentially as previously described^42^,^43^.

### Reformatting of scFv to IgG

Antibodies were converted from scFv to IgG format by subcloning the VH and VL domains into human IgG heavy chain and light chain expression vectors based on those originally described by Persic *et al*.^44^ with an additional OriP element engineered into each. The plasmids were co-transfected into HEK293/EBNA mammalian cells for expression and IgG proteins purified from the culture medium using Protein A chromatography.

### High performance size exclusion chromatography (HP-SEC)

MEDI1912 and MEDI1912_STT IgGs were analysed by HP-SEC using an Agilent 1100 series HPLC fitted with a TSK SW_XL_ HPLC guard column (Tosoh Bioscience, cat. 08543) and TSK-GEL G3000SW_XL_ HPLC column (Tosoh Bioscience, cat. 08541). 50 μL of IgG at 1 mg/mL in Dulbecco’s Phosphate Buffered Saline (D-PBS) (Sigma-Aldrich) was injected at a flow-rate of 1 mL/min using 0.1 M sodium phosphate, 0.1 M sodium sulphate, pH 6.8 as the mobile-phase buffer.

### Analytical ultracentrifugation

Sedimentation velocity analytical ultracentrifugation (SV-AUC) of antibody samples was conducted on a Beckman Coulter XL-A instrument (Beckman Coulter, Inc. CA, USA) at 20 °C. MEDI1912 and MEDI1912_STT were dialysed into 150 mM ammonium acetate, pH 6 or analysed in ‘succinate-Arg buffer’ (20 mM sodium succinate, 125 mM arginine, pH 6.0) at the same protein concentration (1 mg/mL). A wavelength of 298 nm and rotor speed of 28,000 rpm were used. Data were analysed using SEDFIT^45^.

### Poly(ethylene glycol) (PEG) precipitation assay

A stock solution of 40% (w/v) PEG-8000 in succinate-Arg buffer was prepared as the titrant. Stock protein solutions were prepared at 9.8 mg/mL in the same buffer. Samples were prepared in triplicate and loaded into a 96–well, clear, flat-bottomed plate (Greiner Bio-One, cat. 655801) using a liquid handling system (Freedom-EVO150, Tecan, Männedorf, Germany) as follows: (i) succinate-Arg buffer was dispensed into each well with volume of 100–180 μL (ii) 20 μL of protein solution was dispensed into each well to aa final concentration of 1 mg/mL; (iii) 0–80 μL of the PEG-8000 stock solution was titrated into each well to give a series of PEG-8000 concentrations from 0–16%; (iv) samples were mixed by slowly aspirating and dispensing the well contents five times (total sample volume in each well was 200 μL). Plates were examined for air bubbles and nephelometry measurements were made immediately using a NEPHELOstar Plus (BMG Labtech, Ortenberg, Germany).

### Viscosity measurements

Protein samples were prepared at 9.8 mg/mL in succinate-Arg buffer. Viscosity measurements were performed in triplicate at 23 °C on a Microfluidic Viscometer/Rheometer-On-a-Chip (mVROC, Malvern, UK) using sensor 14RA05100462 with an applied constant shear rate of 2000 s^−1^.

### MEDI-578 and MEDI1912 serum concentrations in rat and PK analysis

All *in vivo* work was carried out in accordance with UK Home Office ethical and husbandry standards under the authority of an appropriate Project Licence, which was approved by Babraham Institute Animal Welfare and Ethical Review Body (AWERB).

Antibody PK data were determined following intravenous administration of a single dose of the therapeutic antibody in rats or cynomolgus monkeys. Serum samples were prepared from blood collected at various time points. Serum concentrations of MEDI1912 or MEDI1912_STT in Crl:CD (Srague Dawley) rat serum were determined using a sequential flow-through sandwich method using the Gyrolab™ Workstation. The fluorescence-based assay utilised a 3 step capture-analyte-detection Gyrolab™ method in combination with a Bioaffy 200 CD. Commercially obtained mouse anti-human IgG antibodies were used as capture and detection reagents within the assay and labelled with biotin or alexa-647 respectively. Clearance, T max and terminal half-life were determined for MEDI-1912 and MEDI-1912_STT using the validated software package WinNonlin^®^ version 5.3 (Pharsight^®^).

### Hydrogen deuterium exchange mass spectrometry

Hydrogen exchange was performed using an HDX Manager (Waters) equipped with a CTC PAL sample handling robot (LEAP Technologies), essentially as described previously^27^,^46^. Two different protein stock concentrations were used (10 mg/mL and 1 mg/mL), 10-fold diluted in deuterated buffer (20 mM sodium succinate, 125 mM L-arginine, pD 6.0 at 293 K) to yield final concentrations of 1 mg/mL and 0.1 mg/mL that represent majority dimeric and monomeric populations of MEDI1912 IgG, respectively. To enable comparison between mutant IgGs, back-exchange was corrected per peptide, per protein following incubation in deuterated quench buffer at 333 K for 24 h. Deuterium incorporation was measured in DynamX (Waters) and data normalisation was calculated with in-house software written in MatLab (Mathworks) and Python. Structural representations were generated with PyMol (Schrödinger LLC) and plots in Figures 2 and 3a,b and Supplementary Figure 3 prepared with Prism (GraphPad). To assess statistically significant differences between data collected under predominantly monomeric versus dimeric conditions, data acquired at each timepoint were subjected to a pairwise Student’s t-test with a p-value ≤ 0.01. Filtered data were taken forward to assess comparative deuteration. The mean summed deuteration level per amino acid is according to equation (1) where 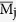 is the mean deuteration level at amino acid *j* summed over the labelling timecourse, *n* is the number of overlapping peptides for amino acid *j, q*_*i*_ is the number of exchangeable amides for peptide species *i*, 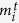 is the isotopic weighted midpoint at time *t* and 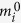 is the midpoint at time 0 (undeuterated).


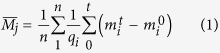


### Protein crystallography

The NGF:scFv complex used for structure determination was produced by incubating human NGF (aa 1–118, expressed and purified by SinoBio (www.SinoBio.net), with the scFv in a 1:1 ratio for 1 h at 4 °C. The complex was subsequently purified by gel filtration on a Superdex 200 10/30 column in 20 mM Tris pH 8, 150 mM NaCl, followed by concentration to 20 mg/mL.

Crystals of the NGF:scFv complex were obtained by sitting drop vapour diffusion at 20 °C by mixing 200 nL of protein (20 mg/mL in 20 mM Tris pH 8, 150 mM NaCl) with 200 nL reservoir solution (18% PEG8000, 150 mM NaCl, 0.1 M Mes, pH 6.5). The crystals appeared spontaneously after >20 days. Crystal-growth rate and crystal quality were improved by seeding. The crystals were cryo protected in the reservoir solution supplemented with 20% (*v/v*) glycerol and subsequently frozen in liquid nitrogen.

Crystallographic data of NGF in complex with the scFv were collected to 3.4 Å resolution at ESRF, Grenoble, at beamline ID23-2 equipped with a Mar225 CCD detector. Data were processed with MOSFLM^47^ and SCALA^48^. The crystal belongs to space group I4, with two full complexes per asymmetric unit. 5% of the reflections were used to calculate *R*_free_.

The structure was determined by molecular replacement using the published structures 1btg^49^ and 1aqk^50^ for NGF and scFv, respectively. The program Phaser^51^ was used to carry out the molecular replacement. Crystallographic refinement was performed with a combination of Refmac5^52^ and autoBUSTER^53^ using NCS restraints. Model building was performed with Coot^54^. Each scFv was divided into two chains with sequence numbering of scFv according to Kabat numbering for antibodies^28^. Data collection and refinement statistics are listed in Supplementary Table 3. The structure factors and coordinates have been deposited with the Protein Data Bank (PDB code 5jz7).

### Structure of the MEDI-578 Fv-NGF dimer complex

We obtained moderate-quality crystals with diffraction up to 3.4 Å, allowing the structure to be determined by X-ray crystallography and to be refined to R_work_ of 25.8% and R_free_ 26.8. Size-exclusion chromatography studies on the MEDI-578/NGF protein complex suggested that the proteins bind in a complex with 2:1 stoichiometry, as confirmed in the solved crystal structure. The structure allowed us to investigate complex formation and analyse interaction interface to the limitations of resolution. Two molecules of MEDI-578 Fv region are bound by single NGF dimer in a unit cell. The two Fv regions bind NGF in a highly similar fashion with almost identical binding contacts. MEDI-578 Fv1 and MEDI-578 Fv2 are extremely similar, with Cα root mean square deviation over 190 residues of 0.2 Å, indicating that there are no major structural differences as a result of their binding to NGF dimer.

### Homology modelling of MEDI-1912 and aggregation prediction

A homology model for MEDI1912 scFv was built using Discovery Studio software from Biovia (formerly Accelrys Software Inc.) starting from a crystal structure of MEDI-578 as a template. Single point mutagenesis was used to generate scFv of MEDI1912. Aggregation-prone regions were predicted using aggregation prediction software from Biovia (formerly Accelrys Software Inc.) and were generated with PyMOL (Schrödinger, LLC).

### Mutagenesis

Based on the aggregation prediction results, the following amino acids in the VH domain of MEDI1912 were mutated to the MEDI-578 sequence by site-directed mutagenesis to create MEDI1912_STT: W30S, F31T and L56T (positions defined by Kabat^28^).

### nESI-MS experiments

Nanoelectrospray nESI-IMS-MS experiments were carried out on a Synapt HDMS instrument (Waters Corp., Wilmslow, Manchester, UK). Samples of MEDI1912 and MEDI1912_STT were dialysed at 1 mg/mL into 150 mM ammonium acetate, pH 6 using dialysis buttons (Hampton Research Corp., Aliso Viejo, CA, USA). nESI-MS experiments were conducted in positive ion mode with samples being introduced using capillaries made in-house. The following instrument settings: capillary voltage 1.5 kV, sample cone 30 V, extraction cone 4 V, source temperature 80 °C, backing pressure 5.0 mBar, trap voltage 40 V, trap/transfer gas flow 1.5 mL/min, IMS gas flow 20 mL/min, IMS ramped wave height 5–30 V, travelling wave speed 300 ms. The m/z scale was calibrated using aqueous caesium iodide (CsI) cluster ions.

### Transmission electron microscopy

Antibody samples were diluted in 20 mM sodium succinate, 125 mM arginine, pH 6, immediately spotted onto carbon coated EM grids (400 mesh, glow discharged 25 mA for 40 seconds) (Fisher Scientific, Leicestershire, UK) and allowed to absorb for 30 seconds. The grids were then washed with milliQ-H_2_O with blotting on Whatman paper between wash steps, Stained with 1% (w/v) uranyl acetate, air dried under a lamp and imaged on a FEI T12 TEM (FEI Inc., Hillsboro, OR, USA) operating at 120 keV.

### Chemical Cross-linking and in-gel digestion

Antibody samples were dialysed into 100 mM sodium phosphate buffer, pH 7 and cross-linked with a 50:50 mixture of d0-BS3 and d4-BS3 (Thermo Scientific, UK) at a 100x molar excess of cross-linker. The reaction was left for 20 minutes before being quenched with 50 mM Tris. HCl, pH 8. Cross-linked and un cross-linked species were separated usinga SDS-PAGE and the cross-linked bands, along with non-cross-linked controls, were excised for in-gel digestion. The gel pieces were subjected to three repeat rounds of hydration and dehydration with 25 mM ammonium bicarbonate, pH 7.8 and 50% acetonitrile/25 mM ammonium bicarbonate, pH 7.8, respectively. The samples were then reduced with 10 mM DTT and alkylated with 55 mM iodoacetamide. The gel pieces were dehydrated again with 50% acetonitrile/25 mM ammonium bicarbonate, pH 7.8 followed by 100% acetonitrile before being re-hydrated with 0.1 μg/μL trypsin solution and incubated overnight at 37 °C. The digested peptides were recovered by subjecting the gel-pieces to three repeat rounds of dehydration with 60% acetonitrile/5% formic acid. The extracted fragments were then concentrated before injection for LC-MS/MS.

### LC-MS/MS of extracted cross-linked digests and analysis

Peptides were analysed on a nanoAcquity LC system connected on-line to a Synapt G2-Si mass spectrometer (Waters Ltd., Wilmslow, Manchester, UK). 1 μL of peptide sample were injected onto an Acquity M-Class column (C18, 75 μm × 150 mm) (Waters Ltd., Wilmslow, Manchester, UK) and separated by a 1–50% gradient elution of solvent B (0.1% v/v formic acid:acetonitrile) in solvent A (0.1% v/v formic acid in water) over a 60 minute time period at a flow rate of 0.3 μL/min. The instrument was operated in positive mode using collision-induced dissociation for fragmentation of the selected ions. Data dependent MS/MS experiments were conducted in the trap region of the instrument using a 1 second scan with the top four most intense ions being selected over a range of 350–2000 *m/z*. Fragmentation of less abundant ions was achieved through manual inclusion in sequential acquisitions after analysis of the MS data. Data were analysed using the MassLynx software (v4.1).

Manual data analysis was achieved through a comparative approach searching for unique peptides corresponding to the digested dimer band of MEDI1912. Cross-linked peptides were readily identified from the peak doublets formed due to the 4 Da shift formed by using a 50:50 mix of deuterated and non-deuterated cross-linker.

### Immunohistochemistry (IHC)

Immunohistochemical staining was undertaken using frozen human tissue microarrays (TMAs) with either MEDI1912 or MEDI1912_STT human IgG1 re-formatted with a murine Fc region to aid IHC detection. An appropriate murine IgG1 isotype control antibody was included in all experiments.

Frozen tissue sections (3 μm) were fixed by briefly immersing in 10% neutral buffered formalin for 15 secs, followed by rinsing in running tap water. Endogenous peroxidase activity was blocked with 0.3% (v/v) hydrogen peroxide (Sigma H-1009) for 15 mins followed by washing in TBST buffer (0.05 M Tris buffered saline; 0.1% (v/v) Tween). Following incubation in 2.5% (v/v) normal horse serum (Vector Labs) for 20 mins samples were incubated with primary antibodies diluted in TBST for 60 mins. Antibody binding was visualized by incubation with an anti-mouse HRP polymer (Vector labs MP-7402) for 30 mins. Following further TBST washing, sections were incubated with 3’3’-diaminobenzidine (Vector ImmPact DAB SK-4105) for 5 minutes. Sections were counterstained with haematoxylin and permanently mounted using DePex.

MEDI1912 and MEDI1912_STT antibodies were optimized, to identify the appropriate concentrations to be used in the experiment. A positive control, using frozen heart tissue containing a central core of NGF conjugated to activated Sepharose beads and a negative control was produced in an identical manner, using BSA conjugated Sepharose beads. All antibodies were tested at a range of concentrations along with the murine IgG negative isotype control. Three concentrations, 0.36, 0.18, 0.09 μg/mL were selected based on positive control staining, and these were taken forward to profile MEDI1912, MEDI1912_STT and isotype across the frozen normal tissue TMA.

Following IHC, all samples were microscopically assessed for the staining profile of the three antibodies. A qualitative assessment of staining location and intensity was undertaken, noting the differences in staining profile between the antibodies.

### pERK assay

Neuroscreen-1 PC12 cells (Thermo Scientific) were plated at 75,000 cells per well on PDL-coated Greiner 96-well tissue culture plates in Dulbecco’s Modified Eagle Medium: Nutrient Mixture F-12 (Life Technologies). Cells were stimulated by the addition of recombinant human β-NGF (R&D Systems, 5 ng/mL final assay concentration), which had been pre-mixed with anti-NGF or isotype control antibodies. 15 min after addition of the NGF, cell supernatants were rapidly removed and cells lysed in 50 μL of lysis buffer from Phospho-ERK Cellular HTRF Assay Kit (Cisbio, Codolet, France). pERK HTRF assay was run according to manufacturer’s instructions.

### Data analysis

All data are expressed as mean ± SD. Data were analysed using GraphPad PRISM (version 2.0) for PC (GraphPad Sotware Inc., San Diego, CA). Where appropriate, IC50 values were determined for each individual experiment and are shown as geometric mean (with 95% confidence limits).

### Epitope Competition Assay

The specificity of MEDI1912 IgG was established using a Homogeneous Time Resolved Fluorescence (HTRF^®^) epitope competition assay. The assay determines relative cross reactivity by measuring inhibition of biotinylated NGF (in house HEK-EBNA derived), binding to MEDI1912 IgG. Binding of MEDI1912 IgG to biotinylated NGF is detected by FRET between an XL6650 labelled anti-human-Fc antibody (CisBio) that binds MEDI1912 IgG and streptavidin cryptate (CisBio) which binds biotinylated NGF. A panel of NGF related proteins (NT-3; NT-4; BDNF; murine NGF; and rat NGF (R&D Systems)) were titrated in the assay and any proteins which bound to MEDI1912 IgG caused a dose dependent decrease in FRET.

### Baculovirus ELISA

Baculovirus particles were produced and used as an antigen for ELISA essentially as previously described by Hotzel *et al*.^5^, with the exception that an anti-human IgG (Fc-specific) HRP-labelled antibody from Sigma (A-0170) was used for detection of antibodies binding to baculovirus particles.

## Additional Information

**How to cite this article:** Dobson, C. L. *et al*. Engineering the surface properties of a human monoclonal antibody prevents self-association and rapid clearance *in vivo. Sci. Rep.* **6**, 38644; doi: 10.1038/srep38644 (2016).

**Publisher's note:** Springer Nature remains neutral with regard to jurisdictional claims in published maps and institutional affiliations.

## Supplementary Material

Supplementary Information

## Figures and Tables

**Table 1:** Viscosity measurements for MEDI1912 and MEDI1912_STT.

	Viscosity, cP		
	20 mM NaSuccinate 125 mM Arg-HCl pH 6	MEDI1912 [9.8 mg/ml]	MEDI1912_STT [9.8 mg/ml]
Test 1	1.044	7.049	1.147
Test 2	1.045	6.876	1.196
Test 1	1.044	6.697	1.192
Avg	1.04	6.87	1.18
Stdev	0.00	0.18	0.03
%CV	0.06%	2.56%	2.31%

**Table 2:** HP-SEC column interaction of MEDI1912 variants.

Antibody	Mutations	HP-SEC monomer retention time (mins)
Reference IgG	N/A	8.7
MEDI-578	N/A	8.7
MEDI1912 (WFL)	—	15.0
Mutant 1 (WFT)	L56T	12.3
Mutant 2 (WTL)	F31T	10.3
Mutant 3 (SFL)	W30S	10.3
Mutant 4 (SFT)	W30S, L56T	9.7
Mutant 5 (STL)	W30S, F31T	9.0
Mutant 6 (STT)	W30S, F31T, L56T	8.9
