# Supplementary material for: Engineering the surface properties of a human monoclonal antibody prevents self-association and rapid clearance in vivo
Source: Sci Rep. 2016 Dec 20;6:38644. doi: 10.1038/srep38644 (PMC5171805; doi:10.1038/srep38644)
Supplement: Supplementary Information [file srep38644-s1.pdf]

Engineering the surface properties of a human monoclonal antibody prevents self-association and rapid clearance *in vivo*.

Claire L Dobson[1], Paul W A Devine[2,3], Jonathan J Phillips[4], Daniel R Higazi[1], Christopher Lloyd[1], Bojana Popovic[1], Joanne Arnold[1], Andrew Buchanan[1], Arthur Lewis[1], Joanne Goodman[1], Christopher F van der Walle[1], Peter Thornton[1], Lisa Vinall[1], David Lowne[1#] , Anna Aagaard[5], Lise-Lotte Olsson[5], Anna Ridderstad Wollberg[5,+], Fraser Welsh[1], Theodoros K Karamanos[2,3], Clare L Pashley[2,3], Matthew G Iadanza[2,3], Neil A Ranson[2,3], Alison E Ashcroft[2,3], Alistair D Kippen[1,‡], Tristan J Vaughan[1], Sheena E Radford[2,3*], and David C Lowe[1*].

[1]MedImmune Ltd, Granta Park, Cambridge, CB21 6GH, UK.
[2]Astbury Centre for Structural Molecular Biology, University of Leeds, Leeds, LS2 9JT, UK
[3]School of Molecular and Cellular Biology, University of Leeds, Leeds, LS2 9JT, UK
[4]Department of Chemical Engineering and Biotechnology, University of Cambridge, Cambridge, CB2 3RA, UK
[5]Discovery Sciences, Innovative Medicines and early Development, AstraZeneca, Pepparedsleden 1, Mölndal, 43183, Sweden
[#]Current address: Immunocore Ltd, Abingdon, OX14 4RY, UK
[+]Current address: Uppsala BIO, Uppsala, 75183, Sweden
[‡]Current address: Ipsen, Wrexham, LL13 9UF, UK

*Corresponding authors
Email LoweD@medimmune.com; S.E.Radford@leeds.ac.uk;

SUPPLEMENTARY MATERIAL

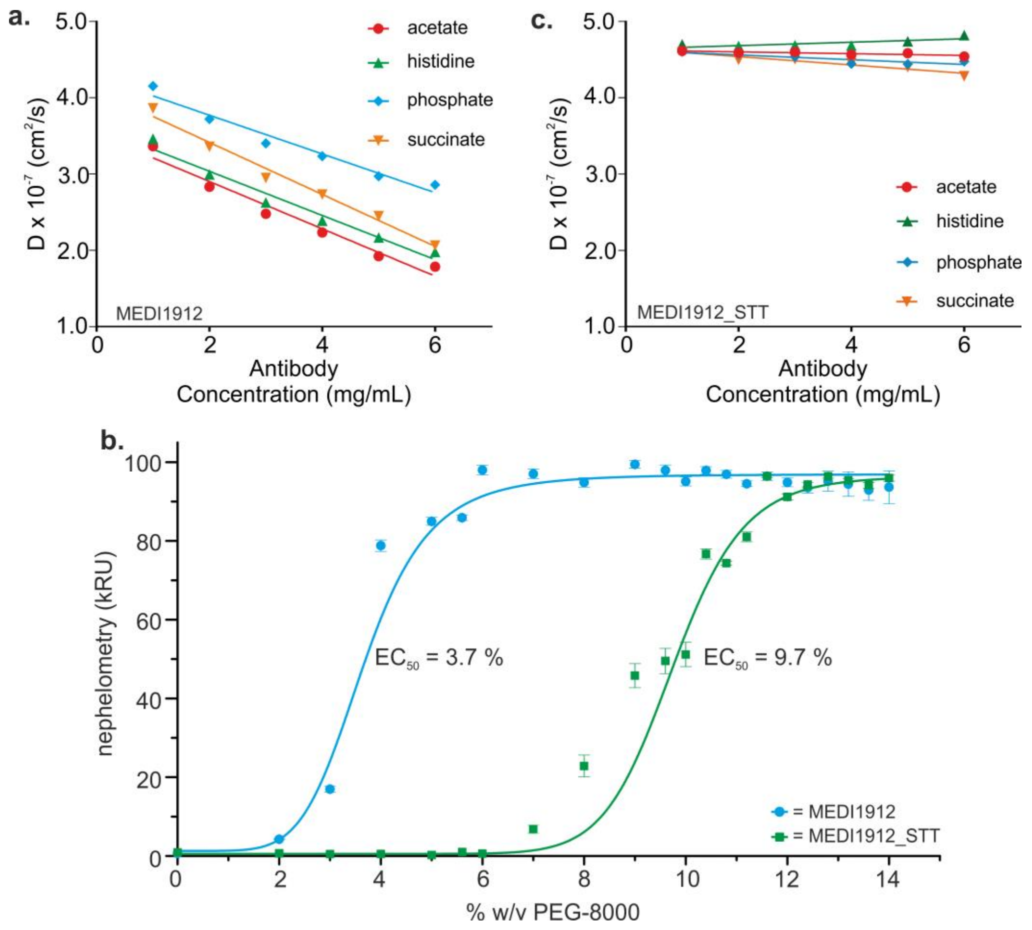

Supplementary Figure 1. Change in diffusion coefficient (*D*) versus concentration of MEDI1912 (a) for four buffer conditions, acetate (10 mM NaOAc/CH_3COOH pH 6), histidine (10 mM His/His.HCl pH 6), phosphate (10 mM Na_2HPO_4/NaH_2PO_4 pH 7.2) and succinate (10 mM NaSuccinate/HCl, pH 6). Relative solubility of MEDI1912 (blue) and MEDI1912_STT (green), as determined in PEG precipitation assay (b). Change in diffusion coefficient (*D*) versus concentration of MEDI1912_STT (c).

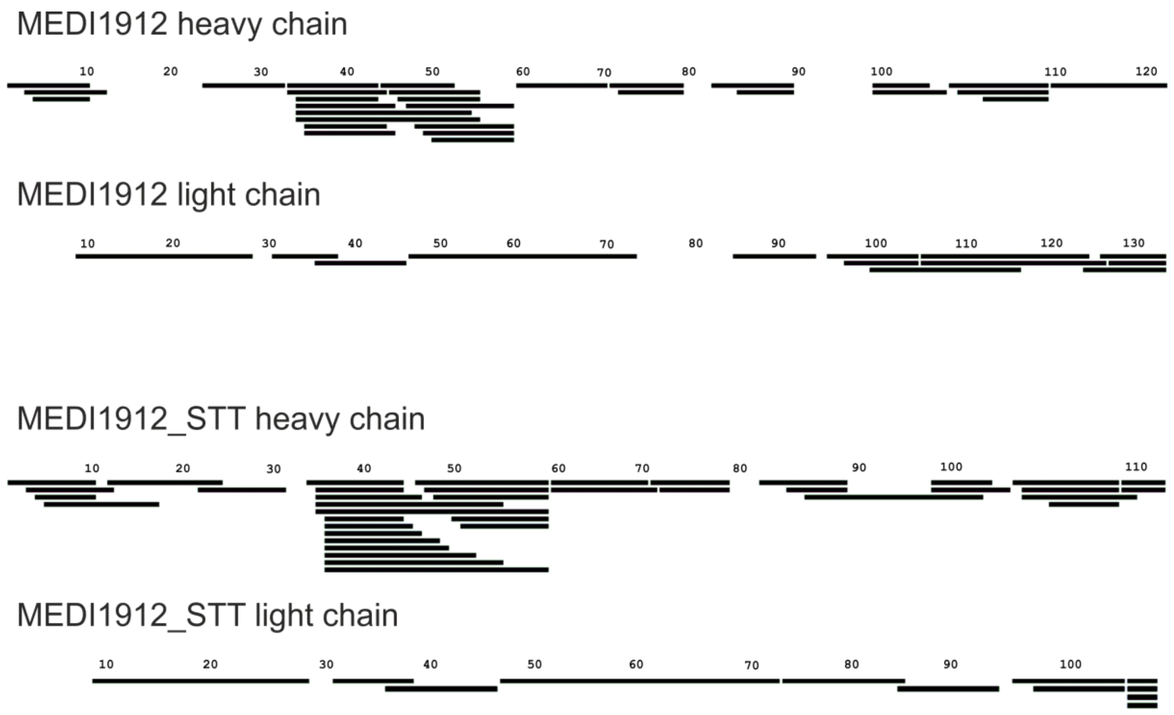

Supplementary Figure 2. Coverage maps for hydrogen/deuterium-exchange mass spectrometry (HDX-MS) of MEDI1912 and MEDI1912_STT variable domains. Each black bar denotes a peptide resulting from pepsin digestion under the conditions used for HDX-MS. Kabat numbering is displayed [1].

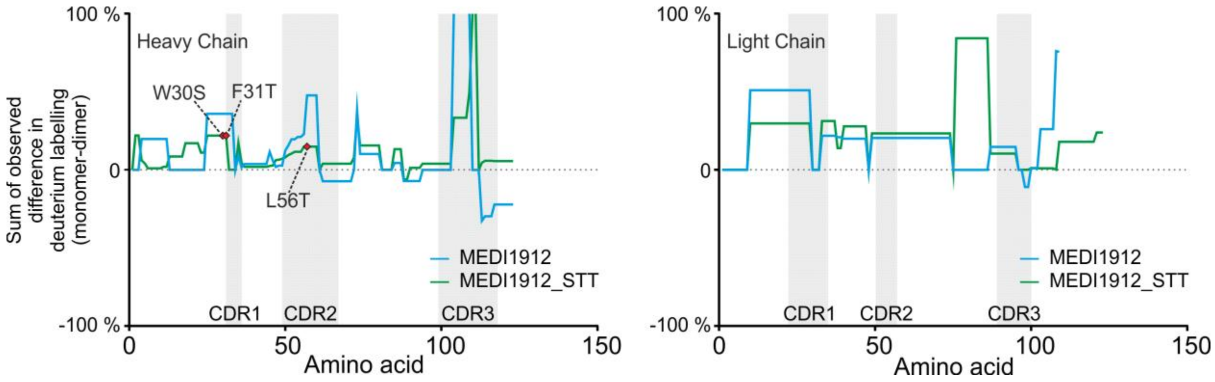

Supplementary Figure 3. Protection against hydrogen-exchange by self-association is reduced in MEDI1912_STT heavy (a) and light (b) chain variable domains. For concentrations at which MEDI1912 is predominantly monomeric (M) and dimeric (D), the difference in uptake of deuterium label was calculated as $\Delta HDX_{(M\text{-}D).}$ This was normalised per protein, with the region that showed maximum difference set to 100%. Protection due to self-association shows as a positive value on the y-axis. The variable domains show a larger difference in HDX protection at the two concentrations investigated for MEDI1912 (blue) than for MEDI1912_STT (green). This supports the conclusion that MEDI1912_STT has reduced self-association by the variable domains and provides site-localised information on the regions responsible for oligomerisation. Grey regions denote CDRs. Three mutations from MEDI1912 to MEDI1912_STT are marked in red, all of which occupy regions with reduced signal of protection due to antibody self-association.

Supp Figure 4

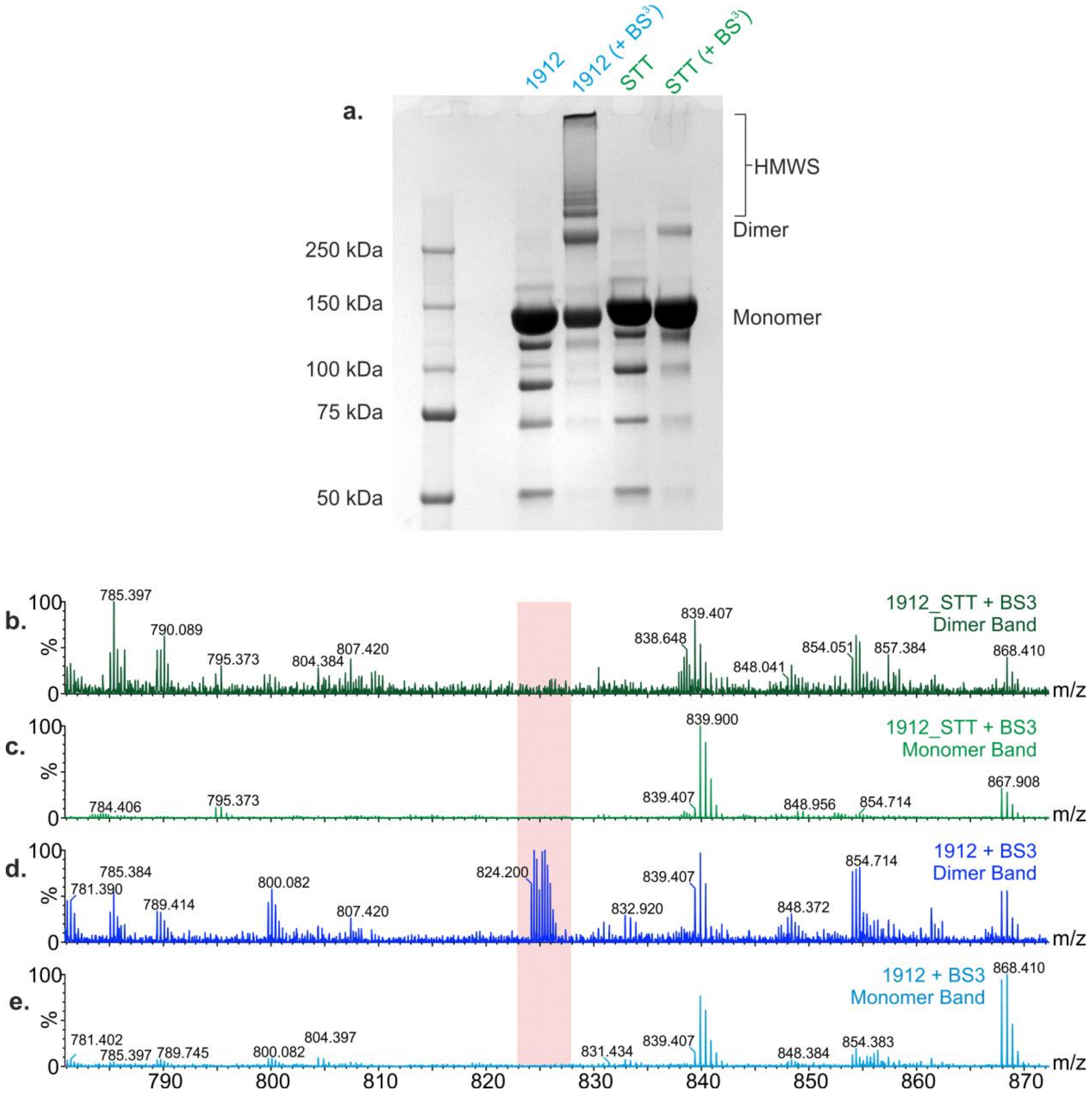

Supplementary Figure 4.

Chemical cross-linking and proteinase digestion reveals a cross-linked peptide that is unique to dimers of MEDI1912. (a) Non-reducing SDS PAGE gel of MEDI1912 and MEDI1912_STT cross linked with BS3 (Methods) HMWS- high molecular weight species. Mass spectra of (b-e) MEDI1912_STT digests from the dimer (b, dark green) and monomer (c, green) bands. (d) MEDI1912 digested dimer band highlighting the unique cross-linked peptide identified at 824.2 *m/z*. (e) MEDI1912 digested monomer band indicating that the peptide identified in (d) is only present in the digested dimer band of MEDI1912.

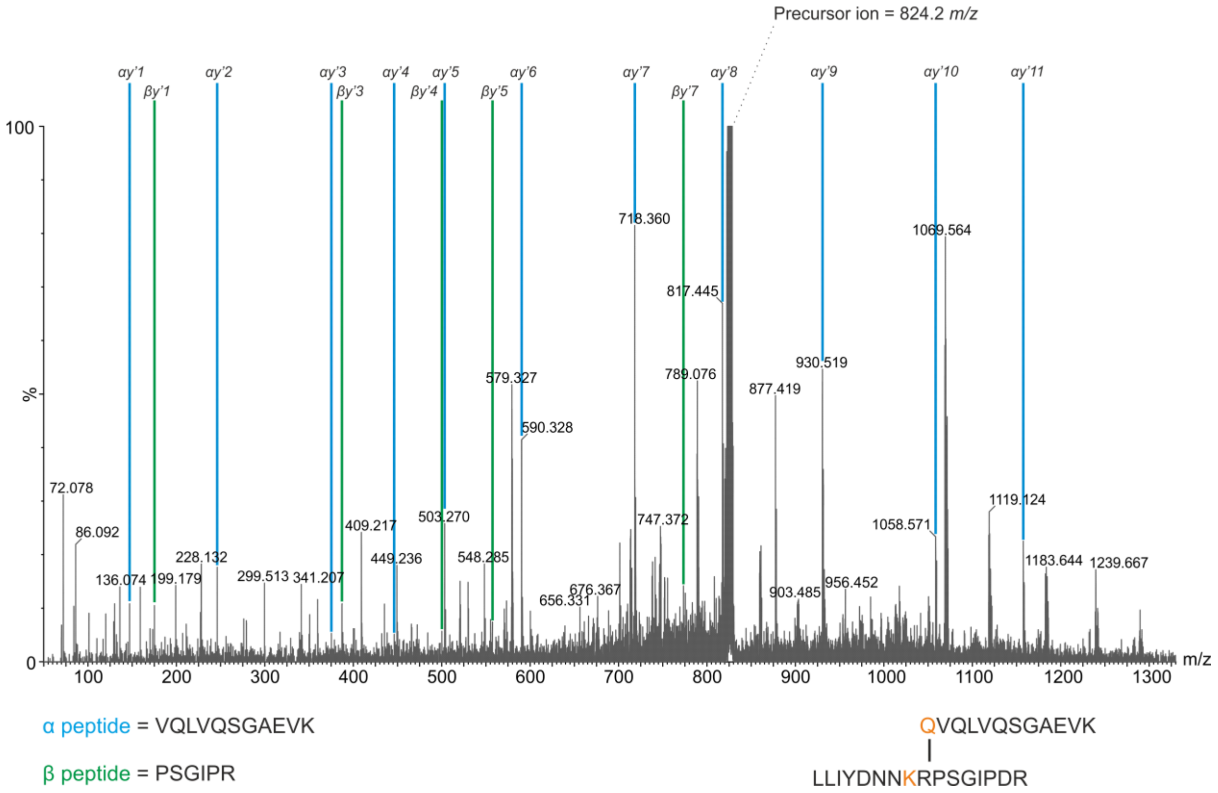

Supplementary Figure 5.

MS/MS sequencing data of the selected peptide (824.2 *m/z*) from the digested dimer band of cross-linked MEDI1912. The two cross-linked peptides are labelled as alpha (blue) and beta (green) with the sequences identified below.

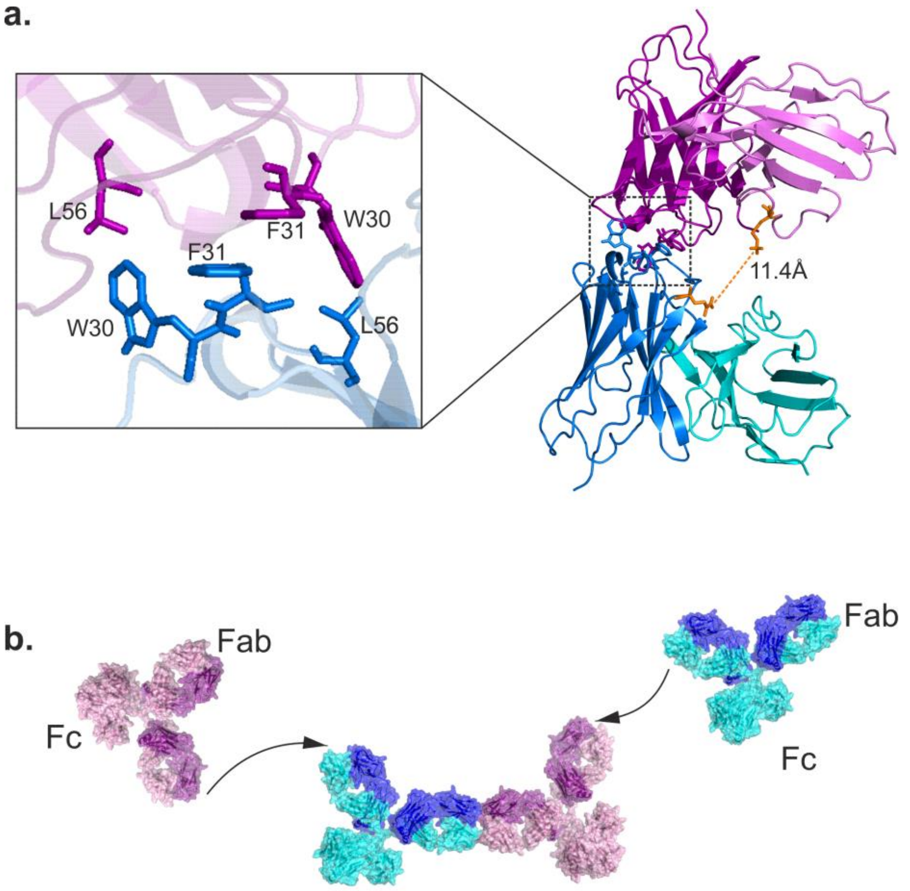

Supplementary Figure 6. Chemical cross-linking to map the binding site of MEDI1912

(a) Chemical cross-linking of MEDI1912 revealed a cross-link between the N-terminus of the heavy chain (Q1) and K54 in the variable region of the light chain. (b) Proposed model of oligomerisation and self-assembly of MEDI1912 via a Fab-Fab interaction consistent with the HX-MS, XL-MS and EM data.

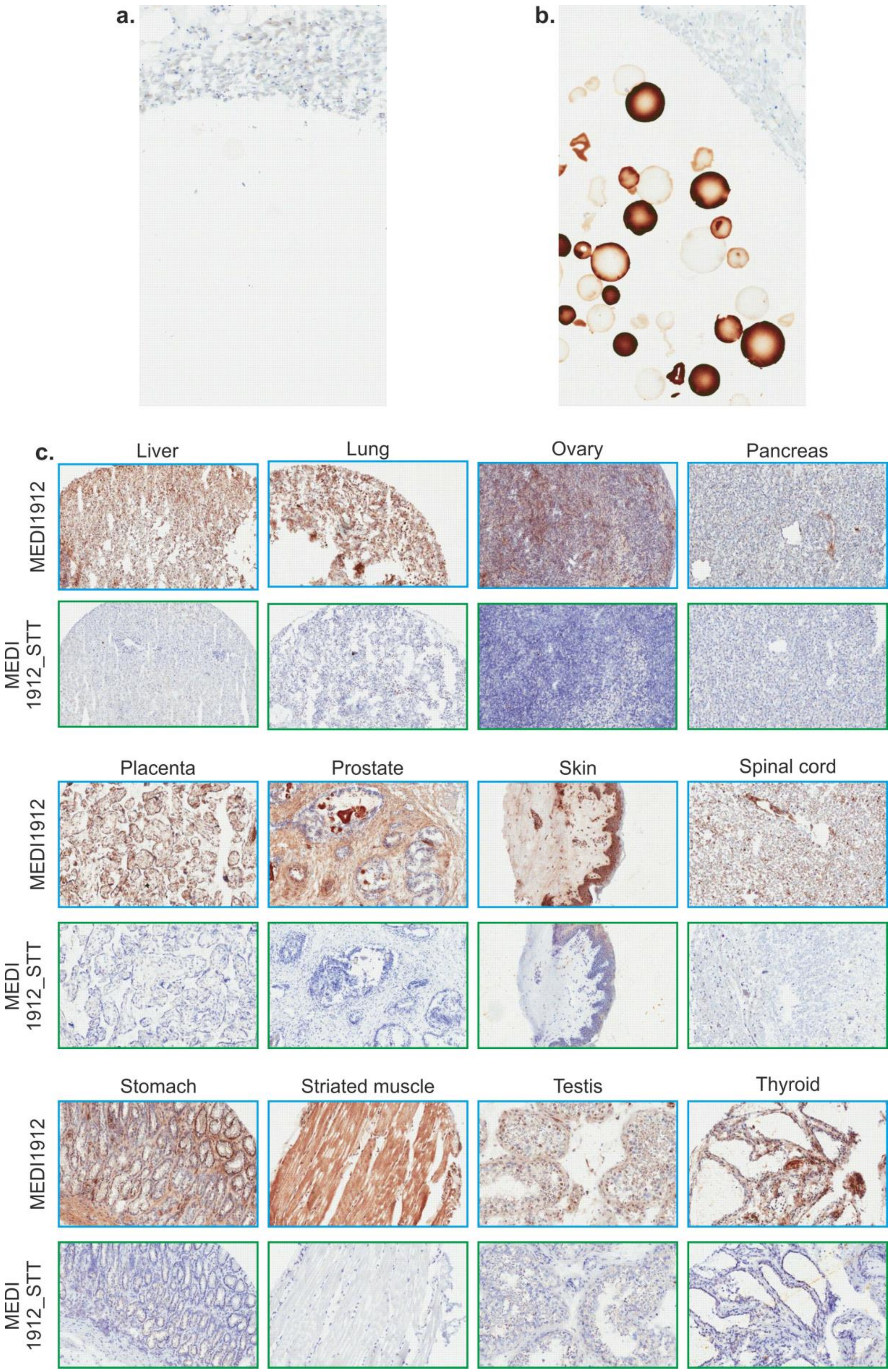
a.
b.
c.
Liver
Lung
Ovary
Pancreas
MEDI1912
MEDI 1912_STT
Placenta
Prostate
Skin
Spinal cord
MEDI1912
MEDI 1912_STT
Stomach
Striated muscle
Testis
Thyroid
MEDI1912
MEDI 1912_STT

Supplementary Figure 7. Heart tissue containing central core of control Sepharose conjugated beads of either (a) BSA or (b) NGF, immunohistochemically stained with MEDI-1912-STT at 0.18 µg/mL. (c) Various human tissue immunohistochemically stained with either MEDI1912_STT or MEDI1912 at 0.18 µg/mL. Significant staining was demonstrated by MEDI1912, showing strong staining in connective tissue, smooth muscle (around blood vessels or in GI tract tissue) and other areas that is consistent with non-specific staining. Isotype control (not shown) showed no evidence of staining in any tissues evaluated.

Supplementary Table 1a. Noncompartmental pharmacokinetic parameters of MEDI-578 and MEDI1912 in rat

Group	Dose (mg/kg)	C_{max} (μg/ml)	AUC $_{(0-\infty)}$ (day*μg/mL)	CL (mL/kg/day)	V_d (mL/kg)	$T_{1/2}$ (days)
MEDI-578	3	45.34	426.00	7.07	166.5	12.09
MEDI-578	0.3	5.44	36.63	8.21	130.5	11.78
MEDI1912	3	41.7	121.4	25.11	124.3	3.70
MEDI1912	0.3	3.78	9.60	31.6	142.7	3.41
MEDI1912	0.03	0.28	0.50	61.47	173.1	2.21

Supplementary Table 1b. Noncompartmental pharmacokinetic parameters of MEDI-578 and MEDI1912 in cynomolgus monkey

Group	Dose (mg/kg)	C_{max} (μg/ml)	AUC $_{(0-\infty)}$ (day*μg/mL)	CL (mL/kg/day)	V_d (mL/kg)	$T_{1/2}$ (days)
MEDI-578	1	35.35 (4.05)	214.6 (33.0)	4.74 (0.77)	102 (13.76)	17.88 (1.87)
MEDI1912	1	17.0 (1.92)	61.2 (24.4)	17.9 (5.99)	119 (68.1)	6.35 (6.82)

Supplementary Table 1c. Noncompartmental pharmacokinetic parameters of MEDI1912 and MEDI1912_STT in rat

Group	Dose (mg/kg)	C_{max} (μg/ml)	AUC $_{(0-\infty)}$ (day*μg/mL)	CL (mL/kg/day)	V_d (mL/kg)	$T_{1/2}$ (days)
MEDI1912	3	48.0 (3.77)	122 (7.16)	24.5 (1.40)	143 (13.8)	4.03 (0.40)
MEDI1912_STT	3	65.0 (3.01)	413 (14.9)	7.26 (0.26)	98.7 (4.00)	9.43 (0.40)

AUC = Area under serum concentration time curves; CL = Clearance; Vd = Volume of distribution; $T_{1/2}$ = Elimination half-life; n = 3 animals per group; numbers in parentheses represent standard deviation.

Supplementary Table 2. Affinity of MEDI1912 and MEDI1912_STT determined by BIAcore

Antibody	Off-rate (1/s)	Kobs on-rate (1/Ms)	K_d range (pM)
MEDI1912	2.0e-4 to 3.3e-5	2.1e7	9.8 to 1.6
MEDI1912_STT	1.5e-4 to 3.2e-5	1.8e7	8.3 to 1.8

Supplementary Table 3. Data collection and refinement statistics

**Data collection**	
Space group	I4
Unit cell dimensions	
(Å)	a=182.1, b=182.1, c=109.8
(°)	α=90.0, β=90.0, γ=90.0
Resolution range (Å)[a]	47.02-3.40 (3.49-3.40)
No. of observations	178760
No. of unique reflections	24799
Data redundancy[a]	7.2 (6.8)
Data completeness (%)[a]	99.9 (99.7)
$< I/\sigma(I) >$[a]	5.3 (1.9)
R_{merge} [a]	0.37 (1.10)
**Refinement**	
Resolution range (Å)[a]	47.02-3.40 (3.49-3.40)
R_{work} (%)[a]	25.8 (36.8)
R_{free} (%)[a]	26.8 (41.5)
Wilson *B*-factor ($Å^2$)	39.9
Overall mean *B*-factor ($Å^2$)	56.9
No. of atoms	
Protein atoms	10556
Heterogen atoms	0
Solvent atoms	0
r.m.s.d. values	
Bond lengths (Å)	0.007
Bond angles (°)	0.90

[a] Numbers in parentheses refer to the highest resolution shell.

SUPPLEMENTAL METHODS

Dynamic Light Scattering (DLS)

DLS measurements were made using a Wyatt DynaPro PlateReader II (Wyatt, Santa Barbara, CA) with a laser wavelength of 820.17 nm. Three independent samples were prepared for each mAb and buffer condition and 30 µL loaded into wells on a 384 well black non-treated polystyrene plate (Thermo Scientific Nunc, UK) over a protein concentration range of 1-6 mg/mL. For each well, ten DLS measurements of 5 s each were acquired at 20 °C and the data were discarded if the percent polydispersity was > 15 %. Cumulants analysis was performed using the Wyatt Dynamics Software ver 7.1.7.16 according to the method of Koppel [2], to directly measure the protein self-diffusion coefficient (D) in the three samples for each condition, which were then averaged. Since D scales with protein concentration (c) according to the equation: $D = D_0(1 + k_D.c)$, where D_0 is the protein diffusion coefficient at infinite dilution, the protein-protein interaction parameter, k_D, can be determined from a plot of D vs c.

Affinity determination by surface plasmon resonance (SPR)

All affinity and kinetic measurements were performed using surface plasmon resonance technology of a Biacore 2000 instrument in HBS-EP buffer (Biacore). For affinity measurements, 100 RU MEDI1912 or MEDI1912_STT IgG were amine coupled to a C1 sensor chip (Biacore). Two-fold serial dilutions of recombinant βNGF (R&D Systems) (10 nM to 78 fM) in HBS-EP buffer (Biacore) were passed over the chip at 50 µl/min (300-3600 s dissociation). Kinetic data were fit to a 1:1 Langmuir binding model.

Generation of a Dimer Model

The MEDI1912 homology model (from MEDI-578) was submitted to HADDOCK [3] with W30, F31 and L56 set as the active residues to drive the docking. The best scoring HADDOCK model was then refined in XPLOR-NIH [4] using a distance restraint of 11.4 Å between residues 1 and 198 with a square well energy potential and residues W30, F31 and L56 as sparse, highly ambiguous distance restraints[5].

REFERENCES

1. Kabat, E. A. & Wu, T. T. Identical V region amino acid sequences and segments of sequences in antibodies of different specificities. Relative contributions of VH and VL genes, minigenes, and complementarity-determining regions to binding of antibody-combining sites. *J. Immunol.* **147**, 1709-1719 (1991).

2. Koppel, D. E. Analysis of macromolecular polydispersity in intensity correlation spectroscopy: the method of cumulants. *Journal of Chemical Physics* **57**, 4814-4820 (1972).

3. de Vries, S. J., van Dijk, M. & Bonvin, A. M. The HADDOCK web server for data-driven biomolecular docking. *Nat. Protoc.* **5**, 883-897 (2010).

4. Schwieters, C. D., Kuszewski, J. J., Tjandra, N. & Clore, G. M. The Xplor-NIH NMR molecular structure determination package. *J. Magn. Reson.* **160**, 65-73 (2003).

5. Clore, G. M. & Schwieters, C. D. Docking of protein-protein complexes on the basis of highly ambiguous intermolecular distance restraints derived from 1H/15N chemical shift mapping and backbone 15N-1H residual dipolar couplings using conjoined rigid body/torsion angle dynamics. *J. Am. Chem. Soc.* **125**, 2902-2912 (2003).
